# Supplementary figures and images for: The detailed distribution of T cell subpopulations in immune-stable renal allograft recipients: a single center study
Source: PeerJ. 2019 Feb 8;7:e6417. doi: 10.7717/peerj.6417 (PMC6369828; doi:10.7717/peerj.6417)

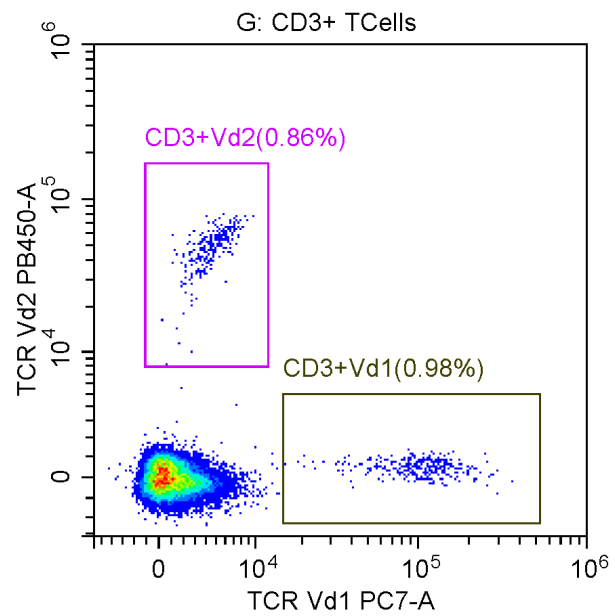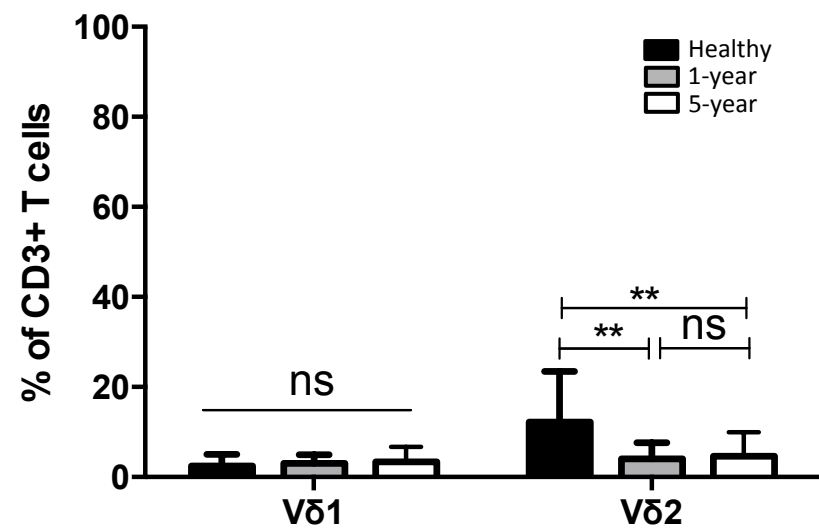

Supplement: Supplemental Information 1 — CD3+ Vδ1 T cells (healthy:2.48±2.59, 1-year:3.00±1.96, 5-year:3.37±3.30); CD3+ Vδ2 T cells (healthy:12.24±11.23, 1-year: 4.05±3.55, 5-year:4.60±5.37). Data are expressed as mean number of each group (mean±SD). **p<0.01. [file peerj-07-6417-s001.pdf]

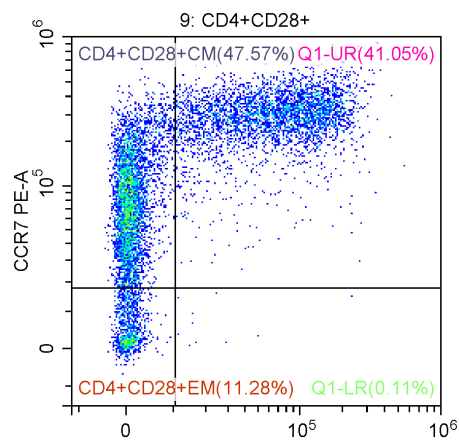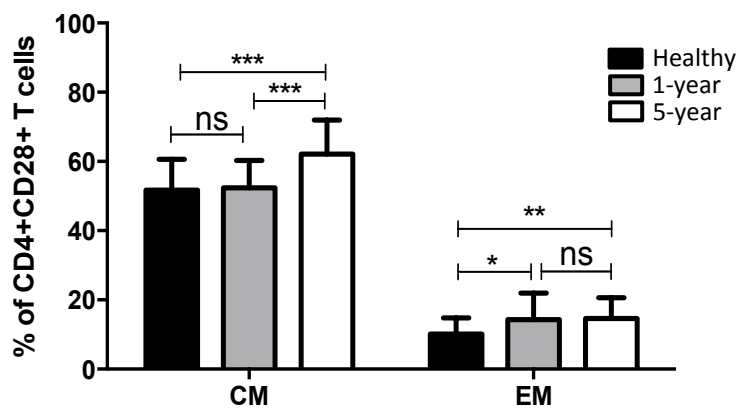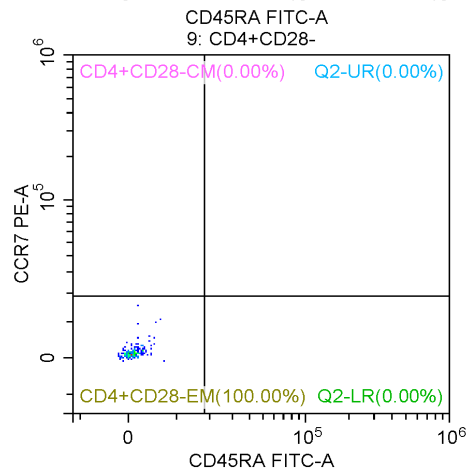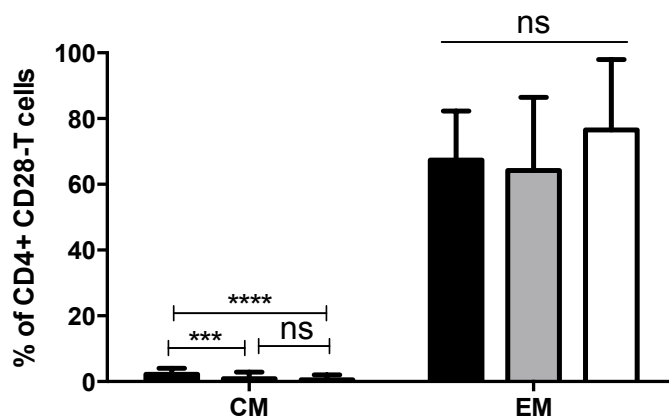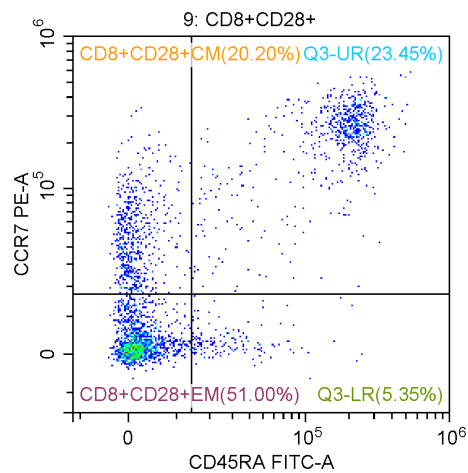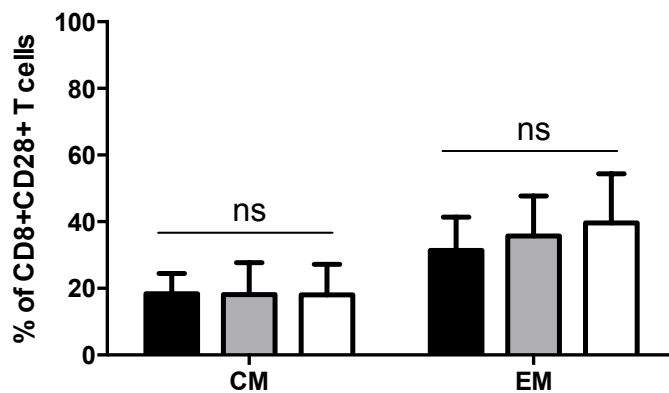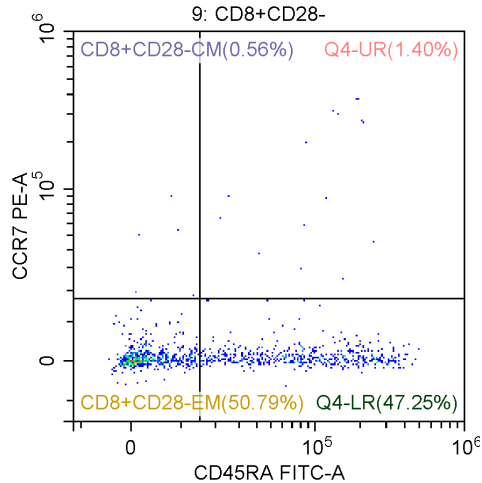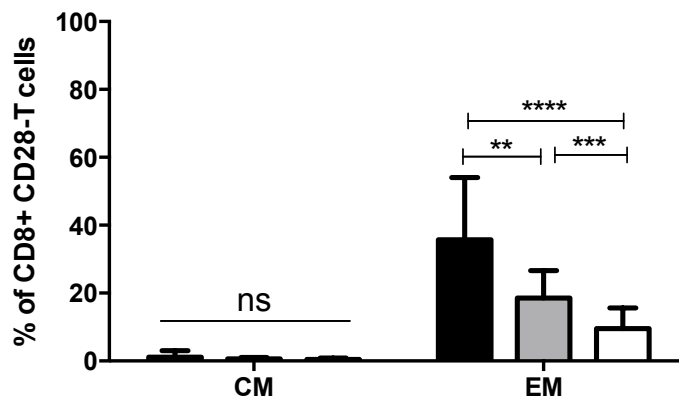

Supplement: Supplemental Information 2 — CD4+CD28+ CM T cells (healthy:51.68±8.98, 1-year:52.39±7.93, 5-year:62.12±9.84); CD4+CD28+ EM T cells (healthy:10.13±4.68, 1-year:14.31±7.66, 5-year: 14.59±6.07); CD4+CD28- CM T cells (healthy:2.19±1.81, 1-year:0.84±2.01, 5-year:0.56±1.51); CD4+CD28- EM T cells (healthy:67.36±14.96, 1-year:64.24±22.21, 5-year:76.50±21.44); CD8+CD28+ CM T cells (healthy:18.40±6.09, 1-year:18.12±9.57, 5-year:18.06±9.12); CD8+CD28+ EM T cells (healthy:31.39±9.96, 1-year:35.73±12.01, 5-year:39.65±14.75); CD8+CD28- CM T cells (healthy:1.41±1.93, 1-year:0.59±0.44, 5-year:0.46±0.41); CD8+CD28- EM T cells (healthy:35.67±18.36, 1-year:18.58±8.02, 5-year:9.53±6.07). Data are expressed as mean number of each group (mean±SD). *p<0.05, **p<0.01, ***p<0.001, ****p<0.0001. [file peerj-07-6417-s002.pdf]

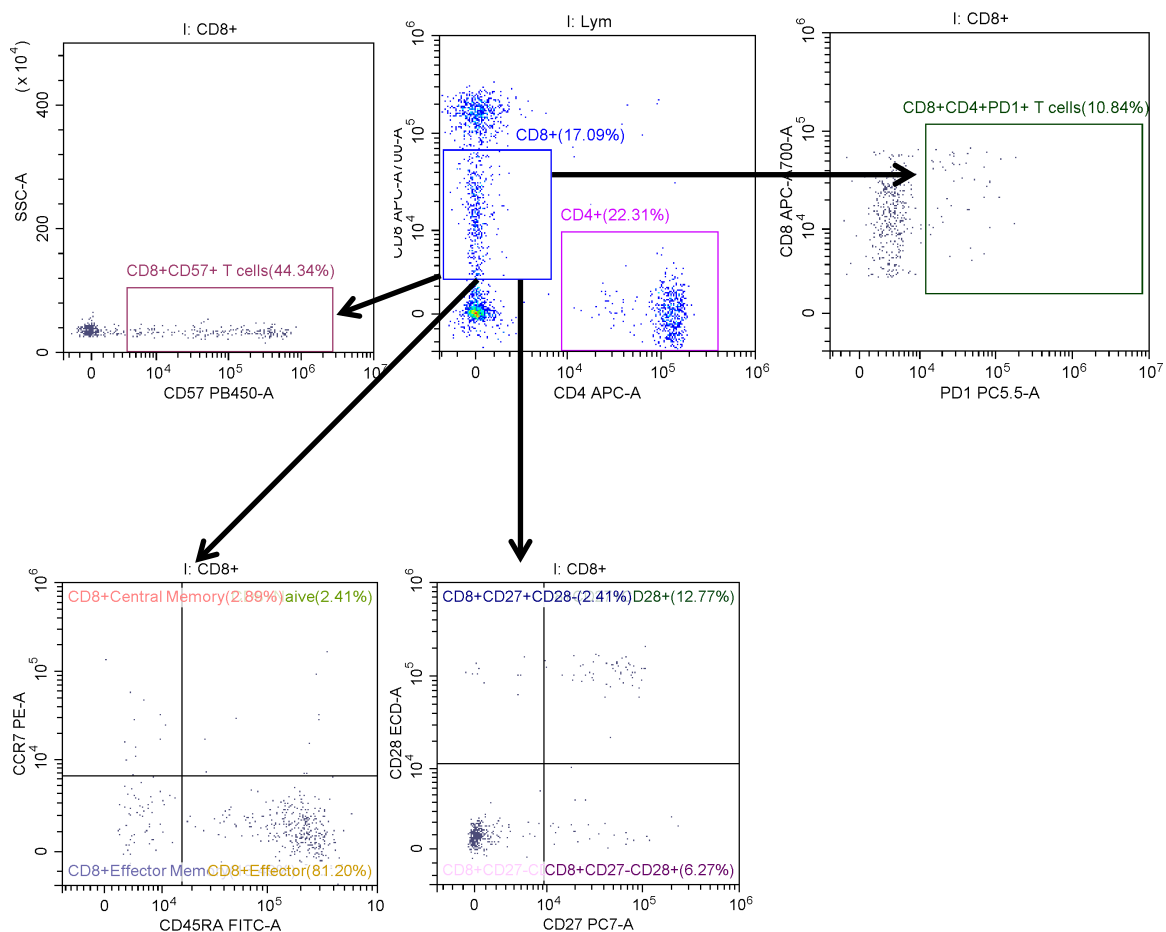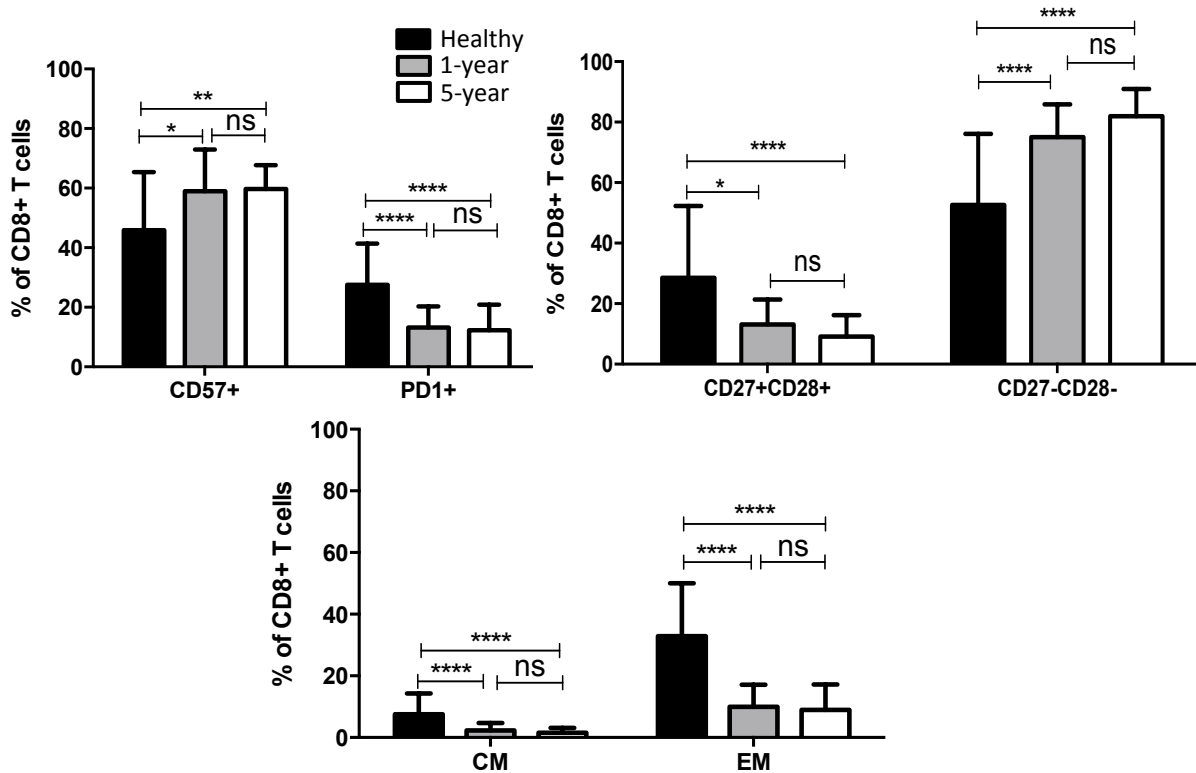

Supplement: Supplemental Information 3 — CD8lowCD57+ T cells (healthy:45.88±19.50, 1-year:58.95±14.02, 5-year:59.69±8.02); CD8lowPD1+ T cells (healthy:27.52±14.84, 1-year:13.17±7.14, 5-year:12.27±8.63); CD8lowCD27+ CD28+ T cells (healthy:28.52±23.79, 1-year:13.09±8.25, 5-year:9.16±7.01); CD8lowCD27- CD28- T cells (healthy:52.65±23.47, 1-year:75.04±10.83, 5-year:81.94±9.05); CD8low CM T cells (healthy:7.56±6.72, 1-year:2.30±2.44, 5-year:1.58±1.58); CD8low EM T cells (healthy:32.86±17.14, 1-year:9.93±7.19, 5-year:8.99±8.22); data are expressed as mean number of each group (mean±SD). *p<0.05, **p<0.01, ****p<0.0001. [file peerj-07-6417-s003.pdf]

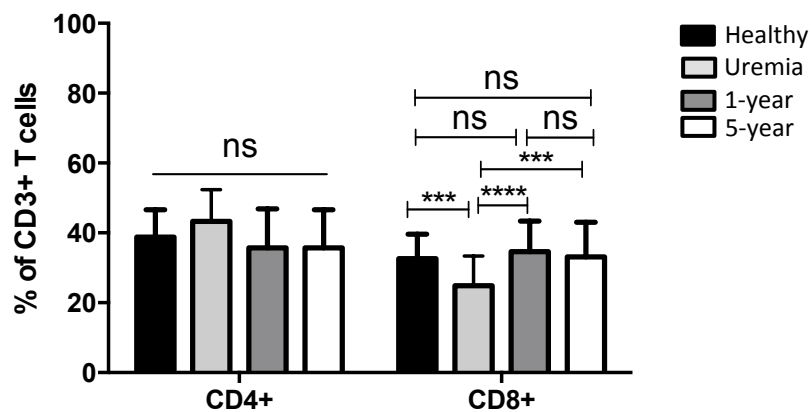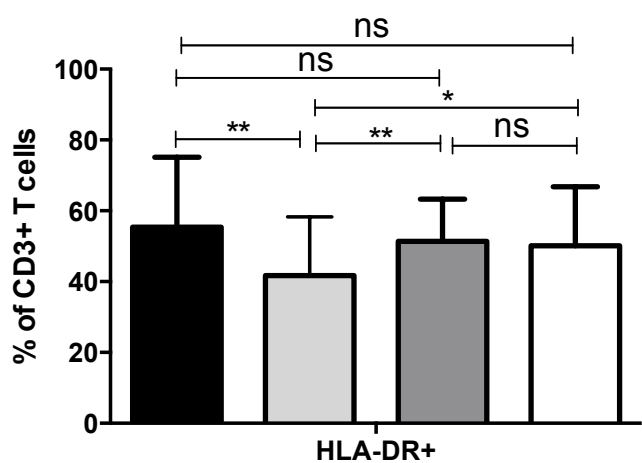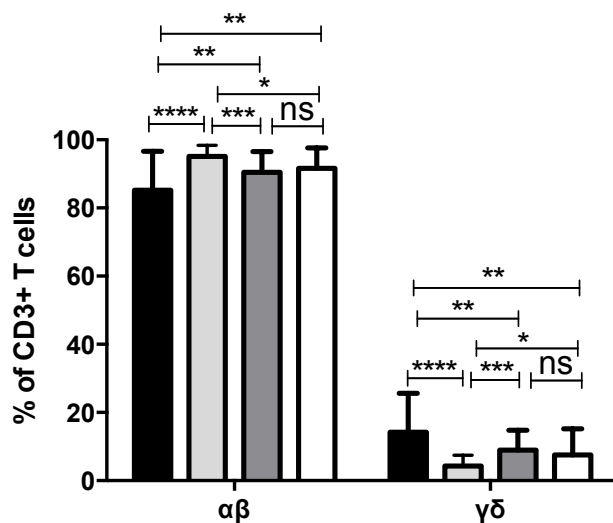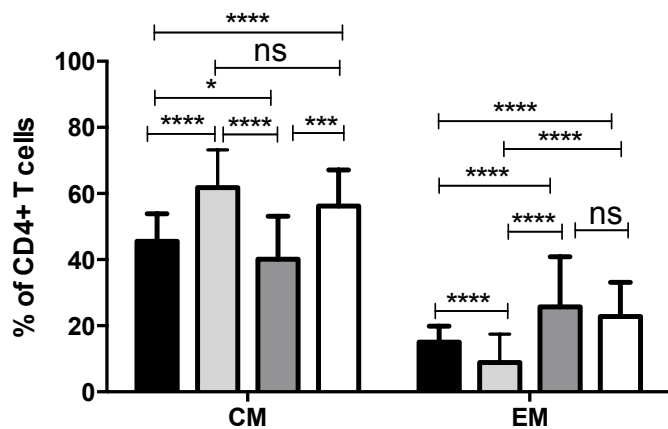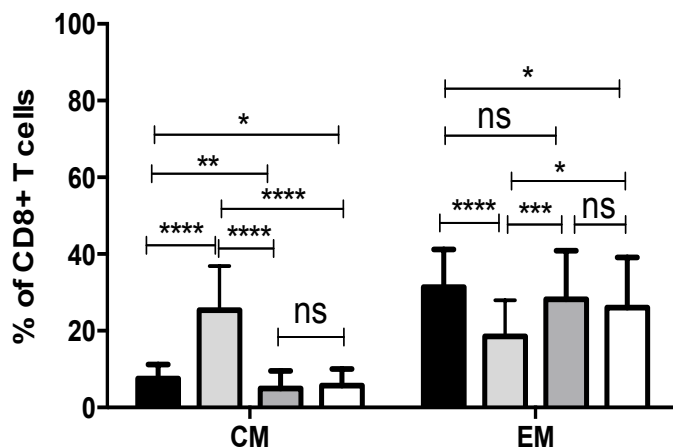

Supplement: Supplemental Information 4 — CD3+CD4+ T cells (healthy:38.77±7.89, uremia: 43.26±9.09, 1-year:35.73±11.15, 5-year:35.03±10.91); CD3+CD8+ T cells (healthy:32.63±6.97, uremia: 24.86±8.49, 1-year:34.64±8.72, 5-year:33.15±9.88); CD3+HLA-DR+ T cells (healthy:55.38±19.77, uremia: 41.71±16.57, 1-year:51.37±11.91, 5-year:49.28±16.09); CD3+ αβ T cells (healthy:85.22±11.41, uremia: 95.10±3.28, 1-year:90.46±6.09, 5-year:91.25±6.16); CD3+ γδ T cells (healthy:14.20±11.39, uremia: 4.32±3.11, 1-year:8.99±5.82, 5-year:8.04±6.04); CD4+ CM T cells (healthy:45.53±8.38, uremia: 61.82±11.39, 1-year:40.13±12.96, 5-year:56.23±10.87); CD4+ EM T cells (healthy:15.02±4.82, uremia: 8.84±8.60, 1-year:25.74±15.14, 5-year:22.76±10.40); CD8+ CM T cells (healthy:7.56±3.66, uremia: 25.41±11.46, 1-year:4.93±4.59, 5-year:5.71±4.29); CD8+ EM T cells (healthy:31.37±9.86, uremia: 18.55±9.39, 1-year:28.17±12.71, 5-year:26.07±13.08). Data are expressed as mean number of each group (mean±SD). *p<0.05, **p<0.01, ***p<0.001, ****p<0.0001. [file peerj-07-6417-s004.pdf]
